# Development of a Discriminant Function Model for Sex Determination From the Anterior Border of the Hip Bone: A Pilot Study on a North Indian Sample

**DOI:** 10.7759/cureus.97826

**Published:** 2025-11-26

**Authors:** Deepak Sharma, Prakarti, Shiv Prakash Sharma, Shatrughan Kumar

**Affiliations:** 1 Anatomy, Rajasthan University of Health Sciences - College of Medical Sciences, Jaipur, IND; 2 Preventive Medicine, Rajasthan University of Health Sciences - College of Medical Sciences, Jaipur, IND

**Keywords:** discriminant analysis, forensic anthropology, hip bone, north indian population, osteometry, sexual dimorphism

## Abstract

Introduction: The hip bone is the most sexually dimorphic element of the human skeleton, crucial for forensic identification. This pilot study aims to develop a preliminary discriminant function model using metric parameters of the anterior border of the hip bone for sex determination in a contemporary North Indian sample.

Methods: An observational study was conducted on 88 adult human hip bones (44 male, 44 female) sourced from two medical colleges in Jaipur, India. Eight linear parameters were measured using digital Vernier calipers. The sample was randomly split into a training set (n=62) for function development and a validation set (n=26). Independent samples t-test identified significant variables, which were then incorporated into a stepwise discriminant function analysis to generate a preliminary sex classification model.

Results: All eight metric parameters demonstrated significant sexual dimorphism (p < 0.001). The stepwise analysis selected three variables: the straight distance from the anterior superior iliac spine to the symphysial surface (ASIS-SS), the straight distance from the anterior inferior iliac spine to the pubic tubercle (AIIS-PT), and the arch distance from the anterior superior iliac spine to the symphysial surface (Arch ASIS-SS). The derived discriminant function achieved a classification accuracy of 95.2% (59/62) in the training set and 92.3% (24/26) in the validation set.

Conclusion: These initial findings suggest that the anterior border of the hip bone provides reliable metric indicators for sex determination in this specific sample. The developed function shows promise as a potential tool for forensic anthropologists working with North Indian skeletal remains, but its generalizability requires validation on larger, more diverse samples.

## Introduction

The identification of sex from human skeletal remains is a cornerstone of forensic anthropology and bioarchaeological inquiry. Among all skeletal elements, the hip bone, or os coxae, is universally regarded as the most reliable indicator due to its pronounced sexual dimorphism, which is fundamentally linked to functional adaptations for parturition in females [1]. These morphological differences are so distinct that sex can often be determined through visual assessment of traits such as the greater sciatic notch, the subpubic angle, and the preauricular sulcus [2]. However, non-metric or morphological methods are inherently subjective, and their accuracy is heavily dependent on the observer's expertise and experience [3]. In contrast, osteometric analysis provides an objective, reproducible, and statistically verifiable alternative, reducing observer bias and allowing for the calculation of classification probabilities [4].

Extensive research has established the utility of various pelvic measurements for sex estimation, with methods ranging from single measurements to complex multivariate models [5]. While the sciatic notch and pubis have been traditional foci, the specific morphological complex of the anterior border, encompassing landmarks such as the anterior superior iliac spine (ASIS), anterior inferior iliac spine (AIIS), pubic tubercle (PT), and symphysial surface (SS), has received comparatively less attention. This region is particularly valuable as it is robust and often well-preserved in forensic contexts. Pioneering work by Gómez Pellico et al. first systematically documented the biometry of this area, demonstrating its value for sex determination [6]. Subsequent studies, such as that by Sachdeva et al. in a North Indian sample, confirmed the significant sexual dimorphism present in these anterior border metrics [7]. Despite this, there remains a pressing need for population-specific standards due to well-documented geographical and ethnic variations in skeletal anatomy [8,9].

The North Indian sample represents a large demographic for which contemporary, statistically robust osteometric standards are still developing. Therefore, this study aims to develop and validate a population-specific discriminant function model for sex determination using metric analysis of the anterior border of the hip bone. The primary objective is to identify the most dimorphic parameters and combine them into a practical mathematical formula that can be applied with high accuracy in forensic casework within this region.

## Materials and methods

Study design and sample

This observational study was conducted on a sample of 88 dry, adult human hip bones of known sex, comprising 44 male and 44 female specimens. The bones were sourced from the Department of Anatomy at RUHS College of Medical Sciences, Jaipur, and SMS Medical College, Jaipur. The inclusion criteria mandated intact bones with no evidence of pathological changes or traumatic damage. Bones exhibiting arthritic changes or any form of damage that could alter the anatomical landmarks were excluded from the study. Ethical clearance for the study was obtained from the institutional ethics committee.

Data collection

Eight metric parameters of the anterior border of the hip bone were measured on each specimen using a digital Vernier caliper with a precision of 0.1 mm. The measurements included the straight distances from ASIS to SS (ASIS-SS), ASIS to PT (ASIS-PT), AIIS to SS (AIIS-SS), AIIS to PT (AIIS-PT), AIIS to iliopubic eminence (AIIS-IE), and iliopubic eminence to PT (IE-PT), as well as the arch distances from ASIS to SS (Arch ASIS-SS) and from AIIS to IE (Arch AIIS-IE). All measurements were taken by a single observer to minimize inter-observer error.

Statistical analysis

The statistical analysis was performed using Statistical Product and Service Solutions (SPSS, version 23.0; IBM SPSS Statistics for Windows, Armonk, NY). The total sample (N=88) was randomly split into a training set (n=62, 70.5%) for model development and a validation set (n=26, 29.5%) for testing the model's performance. An independent samples t-test was used on the training set to compare all metric variables between males and females. Variables demonstrating significant sexual dimorphism (p < 0.05) were subsequently entered into a stepwise discriminant function analysis (DFA) to derive a classification function. The accuracy of this function was calculated for both the training set and the independent validation set. A p-value of less than 0.05 was considered statistically significant for all tests.

## Results

The descriptive statistics for all eight measurements, stratified by sex for the training set, are presented in Table 1. The independent samples t-test revealed that all measured parameters were significantly larger in males compared to females (p < 0.001 for all variables).

**Table 1:** Descriptive statistics and sexual dimorphism of anterior border measurements in the training set (n=62). SD, Standard Deviation; ASIS, Anterior Superior Iliac Spine; AIIS, Anterior Inferior Iliac Spine; PT, Pubic Tubercle; SS, Symphysial Surface; IE, Iliopubic Eminence. Statistical test: Independent Samples T-Test

Measurement (mm)	Males (n=31), Mean ± SD	Females (n=31), Mean ± SD	t-value	p-value
ASIS-SS	133.8 ± 2.9	129.7 ± 2.1	6.45	<0.001
ASIS-PT	120.1 ± 3.2	114.3 ± 1.5	9.18	<0.001
AIIS-SS	101.2 ± 2.5	98.1 ± 1.3	6.23	<0.001
AIIS-PT	87.3 ± 1.8	82.1 ± 2.0	10.89	<0.001
AIIS-IE	39.2 ± 1.7	37.0 ± 1.2	5.95	<0.001
IE-PT	50.7 ± 1.5	49.3 ± 1.7	3.49	0.001
Arch ASIS-SS	158.2 ± 1.2	155.3 ± 1.5	8.41	<0.001
Arch AIIS-IE	43.1 ± 1.4	40.7 ± 1.5	6.58	<0.001

The stepwise DFA was performed on the training set, which selected three key variables for the final model: ASIS-SS, AIIS-PT, and Arch ASIS-SS. The structure matrix indicated that AIIS-PT had the strongest discriminatory power (canonical correlation = 0.84), followed by ASIS-SS and Arch ASIS-SS. The derived discriminant function was as follows: D = (0.351 × ASIS-SS) + (0.482 × AIIS-PT) + (0.289 × Arch ASIS-SS) - 89.456. A sectioning point of 0 was used, whereby a discriminant score (D) greater than 0 classified the bone as male, and a score less than 0 classified it as female.

The classification results of this function are shown in Table 2. The model demonstrated high accuracy, correctly classifying 95.2% (59/62) of the training set cases and 92.3% (24/26) of the validation set cases.

**Table 2:** Classification accuracy of the discriminant function model for sex determination. The discriminant function was derived from the training set and its performance was tested on a separate validation set. The overall accuracy was 95.2% for the training set and 92.3% for the validation set.

Group	Training Set (n=62)	Validation Set (n=26)
	Correctly Classified, n (%)	Correctly Classified, n (%)
Male	30 (96.8)	12 (92.3)
Female	29 (93.5)	12 (92.3)
Total	59 (95.2)	24 (92.3)

## Discussion

The findings of this pilot study demonstrate that the anterior border of the hip bone is a highly sexually dimorphic structure within our specific sample. The significantly larger dimensions observed in males across all measured parameters are consistent with the general robusticity of the male skeleton and reflect the broader architectural framework of the male pelvis. The stepwise discriminant function analysis strategically selected three key variables for the final model: the straight distance from the ASIS-SS, AIIS-PT, and Arch ASIS-SS. Collectively, these measurements capture the superior and inferior extents of the anterior pelvic girdle, effectively quantifying the overall broader and more robust morphology characteristic of males. The high classification accuracy of our preliminary model in both the training and validation sets is encouraging and suggests potential for forensic application.

Our results corroborate and refine the findings of previous research. The work of Sachdeva et al. in a North Indian sample also highlighted the value of anterior border metrics, and our study provides a refined, multivariate model that enhances classification accuracy by isolating the most powerful predictors [8]. Similarly, the foundational work by Gómez Pellico et al. established the normative values and discriminatory power of these measurements, which our findings confirm as highly relevant in a contemporary North Indian sample [7]. The high classification accuracy of our model, achieving 95.2% in the training set and 92.3% in the independent validation set, underscores its robustness. This performance is comparable to the accuracy of other established metric methods for sex determination from the hip bone, such as those utilizing the acetabulum or ischiopubic index, and may even surpass the reliability of subjective trait analysis in inexperienced hands [4,5,10].

The primary strength of this model lies in its practical application. The required landmarks are easily identifiable on intact or partially preserved hip bones, making the function highly applicable in typical forensic scenarios where fragmentation can be an issue. Furthermore, by providing a population-specific formula, this study addresses the critical need for nuanced forensic standards that account for regional anatomical variation, a principle strongly emphasized in modern forensic practice [8,9]. The model serves as a valuable, objective tool that can be used alongside traditional methods to strengthen osteological profiling.

Limitations

It is crucial to interpret these results within the context of the study's limitations. The primary constraint is the sample size, which, while sufficient for an initial model development, limits the generalizability of our findings. Therefore, the function presented here should be considered a preliminary tool specific to samples anatomically similar to our North Indian sample. Future studies with larger, more geographically diverse samples from across the Indian subcontinent are necessary to validate and refine this model before it can be recommended for broad forensic application.

## Conclusions

This study successfully developed and validated a discriminant function model for sex determination using metric analysis of the anterior border of the hip bone in a North Indian sample. The function, based on three key measurements, proved to be highly accurate and reliable. This objective and population-specific tool provides a valuable asset for forensic anthropologists, anatomists, and archaeologists involved in the identification of skeletal remains from this region. Future research should focus on validating this model on larger, more diverse samples from across the Indian subcontinent and exploring its integration with other osteometric methods.

## References

[1] Bruzek J (2002). A method for visual determination of sex, using the human hip bone. Am J Phys Anthropol.

[2] Walrath DE, Turner P, Bruzek J (2004). Reliability test of the visual assessment of cranial traits for sex determination. Am J Phys Anthropol.

[3] Patriquin ML, Steyn M, Loth SR (2005). Metric analysis of sex differences in South African black and white pelves. Forensic Sci Int.

[4] Guyomarc'h P, Bruzek J (2011). Accuracy and reliability in sex determination from skulls: a comparison of Fordisc® 3.0 and the discriminant function analysis. Forensic Sci Int.

[5] Albanese J (2003). A metric method for sex determination using the hipbone and the femur. J Forensic Sci.

[6] Dixit SG, Kakar S, Agarwal S, Choudhry R (2007). Sexing of human hip bones of Indian origin by discriminant function analysis. J Forensic Leg Med.

[7] Gómez Pellico L, Fernández Camacho FJ (1992). Biometry of the anterior border of the human hip bone: normal values and their use in sex determination. J Anat.

[8] Sachdeva K, Singla RK, Kalsey G (2011). The role of the anterior border of the hip bone in sexual dimorphism: a morphometric study in the North Indian population. Med Sci Law.

[9] Murail P, Bruzek J, Houët F, Cunha E (2005). DSP: a tool for probabilistic sex diagnosis using worldwide variability in hip-bone measurements. Bull Mem Soc Anthropol Paris.

[10] Bašić Ž, Anterić I, Vilović K (2013). Sex determination in skeletal remains from the medieval Eastern Adriatic coast - discriminant function analysis of humeri. Croat Med J.

